# Reference intervals for complete blood count parameters in the Longitudinal Study of Adult Health (ELSA-Brasil): a cross-sectional analysis

**DOI:** 10.1590/1516-3180.2025.3017.09122025

**Published:** 2026-05-22

**Authors:** Nívea Aparecida Almeida, Sandhi Maria Barreto, Letícia Gonçalves Resende Ferreira, Chams Bicalho Maluf, Pedro Guatimosim Vidigal, Roberta Carvalho Figueiredo, Danyelle Romana Alves Rios

**Affiliations:** I Undergraduate Student, Universidade Federal de São João del-Rei (UFSJ), Divinópolis (MG), Brazil.; II Professor, Faculdade de Medicina, Hospital das Clínicas (EBSERH), Universidade Federal de Minas Gerais (UFMG), Belo Horizonte (MG), Brazil.; III Research Assistant, Universidade Federal de São João del-Rei (UFSJ), Divinópolis (MG), Brazil.; IV Professor, Faculdade de Medicina, Universidade Federal de Minas Gerais (UFMG), Belo Horizonte (MG), Brazil.; V Professor, Faculdade de Medicina, Universidade Federal de Minas Gerais (UFMG), Belo Horizonte (MG), Brazil.; VI Professor, Universidade Federal de São João del-Rei (UFSJ), Divinópolis (MG), Brazil.; VII Professor, Universidade Federal de São João del-Rei (UFSJ), Divinópolis (MG), Brazil.

**Keywords:** Complete blood count, Reference intervals, Epidemiology, Complete blood count, Reference intervals, ELSA-Brasil

## Abstract

**BACKGROUND:**

Complete blood count (CBC) is the most frequently requested laboratory test worldwide, providing essential clinical information. In Brazil, many laboratories still use reference intervals (RIs) that are inadequately defined or not representative of the local population. Establishing population-specific RIs is crucial for accurate interpretation, diagnosis, and clinical decision making.

**OBJECTIVE:**

To establish RIs for CBC parameters in a sample of Brazilian adults.

**METHODS:**

This cross-sectional study included 2,417 healthy individuals who participated in the baseline (2008–2010) of the Longitudinal Study of Adult Health (ELSA-Brasil). Venous blood collection and storage were performed according to the procedures established by the Clinical and Laboratory Standards Institute (CLSI) in 2018. CBC was performed in laboratories with laboratory proficiency. The RIs were calculated using the nonparametric method proposed by the CLSI.

**RESULTS:**

The RIs were stratified by sex only for the following parameters: Red blood cells (RBC; × 10^6^/mm^3^): Male (4.4–5.6), Female (3.9–5.1); Hemoglobin (g/dL): Male (13.2–16.7), Female (11.8–14.9); Hematocrit (%): Male (39–49), Female (35.3–44.2); Mean corpuscular hemoglobin concentration (MCHC; g/dL): Male (32.3–35.7), Female (32–35.1); Platelets (× 10^3^/mm^3^): Male (146–319), Female: (170–352). The other parameters were as follows: mean corpuscular volume (MCV) (fL): (79.9–96); mean corpuscular hemoglobin (MCH) (pg): (26.4–32.5); White blood cells (WBC; /mm^3^): (3,700–8,610); Neutrophils (/mm ^3^): (1,666–5,705); Eosinophils (/mm^3^): (23.8–530); Basophils (/mm^3^): (0–112); Lymphocytes (/mm^3^): (1,121–2,824); Monocytes (/mm^3^): (240–751.7).

**CONCLUSION:**

Our results agree with those of other studies that have proposed RIs for CBC parameters. The differences found in MCV, neutrophil and WBC parameters (total population), and MCHC (both sexes) may be due to differences in the study populations, sample sizes, and pre-analytical and analytical study variables.

## INTRODUCTION

 Important diagnostic information can be found during a patient’s anamnesis and physical examination;^1^ however, the definitive diagnosis of many diseases depends on laboratory tests.^2^ The complete blood count (CBC) is the most requested laboratory test worldwide. Important information can be obtained from a small amount of blood in about a minute and with a low probability of error through automation.^1 ,2^ The CBC can be used to extract important quantitative and qualitative data on red blood cells (RBC), hematocrit, hemoglobin, mean corpuscular volume (MCV), mean corpuscular hemoglobin (MCH), mean corpuscular hemoglobin concentration (MCHC), RBC distribution width (RDW), white blood cells (WBC) and their subtypes (lymphocytes, neutrophils, monocytes, eosinophils, and basophils), platelet count, and mean platelet volume (MPV).^3,4^

 In 1935, Villela and Rodrigues^5^ were the first researchers to discuss the need to establish reference intervals (RIs) for CBC parameters to assess the health of the Brazilian population. In Brazil, most laboratories use RIs established by the manufacturers of reagents or hematological analyzers used to determine hematological parameters. Some studies have been performed in the Brazilian population, with limited sample sizes.^6-9^

 The National Health Survey (Pesquisa Nacional de Saúde, PNS) established the first RIs for CBC parameters for the adult Brazilian population.^10^ The RI is of paramount importance in laboratory tests, since it supports health professionals in interpreting the results, caring for patients, supporting diagnoses, establishing prognoses, and guiding appropriate treatments.^11^ The Brazilian population is characterized by considerable miscegenation, and RIs can be influenced by several factors: individual (age, sex, race, socioeconomic status, and physiological state) and ecological (exposure to chemical, physical, and biological agents). Therefore, RIs should preferably be defined for each specific population so that possible differences can be estimated more accurately. In Brazil, laboratories commonly define RIs using a methodology that is not suitable and/or not applicable to the local population.^12^

 The Longitudinal Study of Adult Health (ELSA-Brasil) is a large Brazilian cohort study whose main objective is to investigate the incidence and progression of chronic noncommunicable diseases. Throughout the follow-up period, the participants are subjected to a wide variety of interviews and physical, imaging, and laboratory tests, including CBC. Thus, ELSA-Brasil offers a unique opportunity not only to obtain the RIs for blood counts but also to evaluate, over time, the predictive capacity of these RIs for the development of diseases and the determinants of their changes over time. Therefore, the aim of this study was to establish RIs for CBC parameters in a sample of Brazilian adults. 

## MATERIALS AND METHODS

### Design and study population

 The study population consists of participants from ELSA-Brasil. This cohort included 15,105 participants aged between 35 and 74 years. The main objectives of the study are to investigate the incidence and progression of chronic non-communicable diseases, particularly diabetes and cardiovascular diseases, and identify their determinants in Brazilian adults.^13,14^

 All participants were active or retired civil servants from higher education or research institutions in six Brazilian state capitals. The first baseline examination took place between 2008 and 2010, and the study was completed in four waves (wave 1: 2008–2010; wave 2: 2012–2014; wave 3: 2016–2018; wave 4: 20222024). Ethical approval for the study was granted by the Comissão Nacional de Ética em Pesquisa (CONEP), linked to the Ministério da Saúde (approval date: August 4, 2006; CAAE 0016.1.198.000-06), in addition to approval from the institutional ethics committees of each participating center. The design and conduct of ELSA-Brasil followed international ethical standards for human research, including the principles of the Declaration of Helsinki. All participants provided written informed consent prior to study procedures.^13,14^

**Figure 1:**
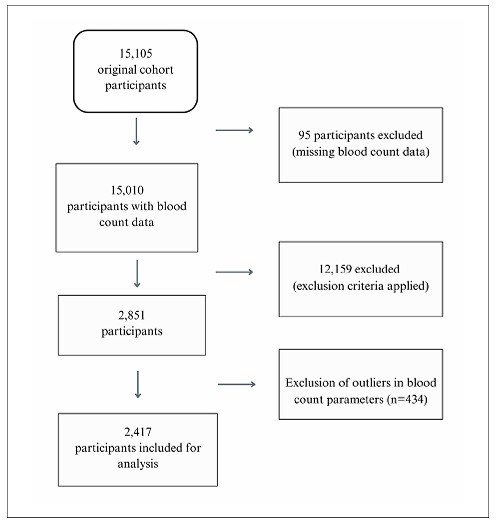
Flowchart of the study population.

 Of the 15,105 participants in the ELSA-Brasil study, 95 were excluded because they lacked CBC data. In addition, participants with factors that could alter their inflammatory status were excluded from the study (**Table S1, supplementary material**). According to the exclusion criteria, of the 15,105 participants, 12,688 were excluded (**Figure 1**), resulting in a sample of 2,417 individuals. 

### Biological samples

 Venous blood was collected in the morning after a 12 h fast and stored at room temperature (18–25^°^C) until the CBC was measured (up to 2 h after collection), according to the procedure established by the Clinical and Laboratory Standards Institute (CLSI) and their guidelines for the collection of diagnostic blood samples by venipuncture and the use of an approved standard.^15^ For the venipuncture technique, a vacuum blood collection system was used, with sample tubes containing the tripotassium salt of ethylenediaminetetraacetic acid (EDTA) identified with a specific barcode for each participant. The tourniquet time did not exceed 1 min. The CBC analysis was conducted locally for technical reasons using automated hematology analyzers in laboratories with internal and external quality controls. The equipments used to conduct the CBC at the local centers were: Bahia and Espirito Santo: MAXM^®^ – Beckman Coulter; Minas Gerais and Rio Grande do Sul: XE 2100 D (Sysmex, Kobe); and São Paulo: CellDyn 3700^®^ Abbott, with hemogram^®^ brand reagents. All laboratories participated in laboratory proficiency programs (the National Quality Control Program, Laboratory Testing Proficiency Program, and College of American Pathologists Accreditation Program).^16^

### Statistical analysis

 The Kolmogorov–Smirnov test was used to evaluate the distribution of the CBC parameters. The RIs were calculated step-by-step, following the CLSI^17^ guidelines. A nonparametric method was used to determine the RIs, which were calculated as the intervals between the 2.5th and 97.5th percentiles of the parameter distribution. The Mann–Whitney U-test and Student’s t-test were used, where appropriate, to evaluate differences between subgroups defined by sex and age. We assessed the need to recommend age- and sex-specific RIs for CBC parameters using the Harris–Boyd statistical approach. Following this approach, we calculated the z-scores of the means and standard deviations (SDs) of the parameters and compared them with the critical value (z^*^). RIs separated by age group and sex were recommended if at least one of the following conditions was met:

1. calculated z exceeds the critical value z^*^;
2. statistical differences exist between the SDs of the hematological parameters of each subgroup and the highest SD exceeds the lowest SD 1.5 times, or if the ratio [highest SD ÷ (highest SD − lowest SD)] is less than 3.

A *p* value of less than 0.05 was considered statistically significant, and the analyses were performed using the statistical package STATA 9.0 (Stata; College Station, Texas). 

## RESULTS

**Figure 2:**
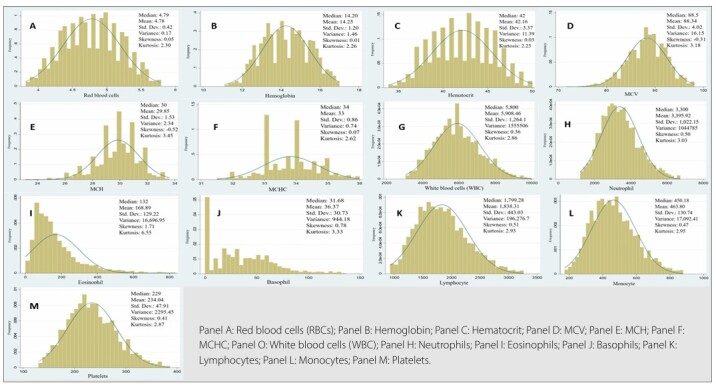
Distribution of hematological parameters in the sample.

 Most participants were enrolled at the São Paulo Study Center (34.4%), female (54.4%), aged 35–59 years (92%), self-declared white (52.6%), and most had completed higher education (57.8%) (**Table 1**). Histograms showing the distribution of hematological parameters are shown in **Figure 2**. 

**Table 1:** Sociodemographic characteristics of the 2,417 reference individuals (ELSA-Brasil, 2008–2010)

**Characteristics**		**Frequency n (%)**
Study Center		
	*São Paulo*	831 (34.4)
	*Minas Gerais*	518 (21.4)
	*Bahia*	357 (14.8)
	*Rio de Janeiro*	301 (12.4)
	*Rio Grande do Sul*	224 (9.3)
	*Espírito Santo*	186 (7.7)
Sex		
	*Female*	1,314 (54.4)
	*Male*	1,103 (45.6)
Age (years)		
	*35–59*	2,223 (92)
	*≥ 60*	194 (8)
Self-reported skin color/race		
	*White*	1,257 (52.6)
	*Brown*	735 (30.7)
	*Black*	310 (13)
	*Other^a^*	89 (3.7)
Schooling (years)		
	*< 11*	187 (7.7)
	*11–14*	832 (34.4)
	*≥ 15*	1,398 (57.8)

a Includes indigenous natives n = 23 (1%), Asian descendants n = 66 (2.8), and missing data n = 26 (1.1%).

 According to the Kolmogorov–Smirnov test, only the MCV showed a symmetrical data distribution, while the other parameters showed an asymmetrical distribution. With respect to sex, most parameters showed a statistically significant difference (p < 0.05), except for MCV, WBC, and lymphocytes. The median values of the RBC count, hemoglobin levels, hematocrit, MCHC, eosinophil and monocyte counts were higher among men, and those of the neutrophil, basophil, and platelet counts were higher among women. In relation to age, a statistically significant difference (p < 0.05) was observed for the MCV; MCH; basophil, lymphocyte, and platelet counts. The results showed higher median values for lymphocytes and platelets in the age group of 35–59 years and for MCV, MCH, and basophils in those aged 60 years and over (**Table 2**). 

**Table 2:** Hematological parameters in relation to sex and age group (n = 2,417) (ELSA-Brasil, 2008–2010)

	**N total(n = 2,417)**	**Sex**			**Age groups**		
		**Male (n = 1,314)**	**Female (n = 1,103)**	**p value**	**Adults (35–59 years) (n = 2,223)**	**Elderly (≥ 60 years) (n = 194)**	**p value**
RBC (× 106/mm^3^)^*^	4.8 (4.5–5.1)	5 (4.8–5.3)	4.5 (4.3–4.7)	< 0.001	4.8 (4.5–5.1)	4.7 (4.5–5)	0.56
Hemoglobin (g/dL)^*^	14.2 (13.3–15.2)	15.1 (14.5–15.7)	13.3 (12.7–13.8)	< 0.001	14.2 (13.3–15.2)	14.4 (13.5–15.2)	0.186
Hematocrit (%)^*^	42 (39.5–45)	44.3 (42.8–46)	39.6 (38–41)	< 0.001	42 (39.5–45)	42 (40–45)	0.286
MCV (fL)^∆^	88.3 (± 4)	88.3 (± 4)	88.4 (± 4.1)	0.367^a^	88.3 (± 4)	89.2 (± 3.8)	0.001^a^
MCH (pg)^* ∆^	30 (29–31)	30 (29–31)	30 (29–31)	< 0.001	30 (29–31)	30.4 (29.3–31)	< 0.001
MCHC (g/dL)^*^	34 (33–34.4)	34 (33.3–34.7)	33.7 (33–34)	< 0.001	33.9 (33–34.4)	34 (33.3–34.5)	0.149
WBC (/mm^3^)	5,800 (5,000–6,700)	5,800 (5,000–6,650)	5,900 (5,000–6,830)	0.318	5,810 (5,000–6,710)	5,700 (4,880–6,480)	0.052
Neutrophils (/mm^3^)	3,300 (2,655–4,026)	3,247 (2,650–3,931)	3,360 (2,673–4,102)	0.013	3,304 (2,668–4,050)	3,202 (2,576–3,843)	0.051
Eosinophils (/mm^3^)^*^	132 (79–218.4)	141.5 (87.3–237.8)	122 (71–195)	< 0.001	132 (78.7–216.8)	138 (89.1–248)	0.163
Basophils (/mm^3^)^* ∆^	31.7 (10.4–56)	30.7 (7–54)	32.6 (13.2–58.8)	0.001	31.3 (9.7–55)	36.6 (18.1–61)	0.016
Lymphocytes (/mm^3^)^∆^	1,799 (1,509–2,112)	1,792 (1,502–2,108)	1,800 (1,518–2,115)	0.636	1,800 (1,515–2,122)	1,704 (1,451–2,042)	0.026
Monocytes (/mm^3^)^*^	450.2 (368.5–547.3)	469.6 (384–561.1)	432 (354–522)	< 0.001	451.5 (368–548)	441.8 (371.4–546)	0.776
Platelets (× 10^3^/mm^3^ )^* ∆^	229 (199–265)	217.5 (190–249)	244 (216–279)	< 0.001	231 (200–267)	221.5 (188–253)	0.003

RBC, red blood cells; MCV, mean corpuscular volume; MCH, mean corpuscular hemoglobin; MCHC, mean corpuscular hemoglobin concentration; WBC, white blood cells. P value obtained using the Mann–Whitney U-test and ^a^t-test; n, sample number. Data are presented as the median (25th–75th percentile) or mean ± standard deviation (SD).

* There was a difference between sex

∆ There was a difference between age groups.

 With respect to sex and age, we found statistically significant differences in the z-scores for the RBC, hemoglobin, hematocrit, MCHC, and platelet parameters. In the analysis by age, no parameter met any of the three conditions of the Harris–Boyd statistical approach for categorizing RIs. Therefore, we chose to present categorized RIs only for parameters that met at least one criterion of the Harris–Boyd statistical approach and CLSI^17^ recommendations. **Table 3** shows the RIs for the stratified hematological parameters. 

**Table 3:** Reference intervals and their respective confidence intervals (95% CI) for hematological parameters (n = 2,417) (ELSA-Brasil, 2008–2010)

**Parameters**		**Median**	**Reference interval**	
			**Percentile 2.5 (95% CI)**	**Percentile 97.5 (95% CI)**
RBC (× 10^6^/mm ^3^)	All	4.8	4 (4–4.05)	5.6 (5.5–5.6)
	Male	5	4.4 (4.3–4.4)	5.6 (5.6–5.7)
	Female	4.5	3.9 (3.9–4)	5.1 (5.1–5.2)
Hemoglobin (g/dL)	All	14.2	12.1 (12–12.2)	16.5 (16.4–16.6)
	Male	15.1	13.2 (13–13.4)	16.7 (16.6–16.8)
	Female	13.3	11.8 (11.6–11.9)	14.9 (14.7–15.1)
Hematocrit (%)	All	42.1	36 (36–36.4)	48.5 (48–49)
	Male	44.3	39 (38.4–39.2)	49 (49–49)
	Female	39.6	35.3 (35–35.8)	44.2 (44–45)
MCV (fL)	All	88.3	79.9 (79–80.2)	96 (95.3–96)
MCH (pg)	All	30	26.4 (26–26.6)	32.5 (32.4–32.9)
MCHC (g/dL)	All	34	32 (32–32.1)	35.5 (35.5–35.6)
	Male	34	32.3 (32–32.6)	35.7 (35.6–35.8)
	Female	33.7	32 (31.9–32)	35.1(35–35.3)
WBC (/mm^3^)	All	5,800	3,700 (3,600–3,790)	8,610 (8,480–8,800)
Neutrophils (/mm^3^)	All	3,300	1,666 (1,570.4–1,716.4)	5,705 (5,609.4–5,822.4)
Eosinophils (/mm^3^)	All	132	23.4 (2.3–32.2)	530 (507.2–566.2)
Basophils (/mm^3^)	All	31.7	0 (0–0)	112 (108–120)
Lymphocytes (/mm^3^)	All	1,799	1,120 (1,092–1,149.7)	2,824 (2,760.3–2,886)
Monocytes (/mm^3^)	All	450.2	240 (234.8–251.3)	751.7 (738.1–776.9)
Platelets (× 10^3^ /mm^3^)	All	229	151 (149–154.4)	339 (332–343.7)
	Male	217.5	146 (140–149)	319 (312–329.3)
	Female	244	170 (164–173.7)	352 (342.3–363.6)

RBC, red blood cells; MCV, mean corpuscular volume; MCH, mean corpuscular hemoglobin; MCHC, mean corpuscular hemoglobin concentration; WBC, white blood cells.

## DISCUSSION

 This study established the RIs for CBC parameters, some of which were stratified according to sex. Men exhibited higher RBC, hemoglobin, hematocrit, and MCHC values than women, corroborating the data from the literature. These differences can be explained by the action of androgen hormones on erythropoiesis and blood loss during the menstrual period in women.^18,19^ However, women had higher platelet counts than men, confirming what the literature indicates, because during menstruation, blood vessels responsible for irrigating the uterine region rupture, causing menstrual bleeding and increasing the number of platelets for stopping the bleeding.^20^

 Our results are similar to those of other studies that have proposed RIs for CBC parameters (**Table S2, supplementary material**). Compared with the RIs reported for other populations, the RIs identified in this study showed a lower upper limit for MCV; slightly higher lower and upper limits in both sexes for MCHC; and lower upper and lower limits in the overall population for WBC and neutrophil counts, both of which exhibited narrower ranges. Pronounced sex-related differences were observed for platelets, with both limits being higher than those commonly reported for international RIs. These discrepancies may reflect variations in population characteristics; sample size; and pre-analytical and analytical factors such as sample collection and processing procedures, analytical techniques, and the use of different hematology analyzers. Collectively, these findings highlight the importance of establishing RIs tailored to the Brazilian adult population. In terms of sample size, the PNS collected data from 8,952 individuals, whereas this study analyzed data from 2,417 participants. The PNS reported that there was no strict schedule for blood collection; the blood was collected at home and processed in local laboratories. The exclusion criteria used by the PNS were pregnant women and individuals diagnosed with serious or chronic illnesses such as cardiovascular disease myocardial infarction, angina, stroke, cancer, arthritis, and chronic kidney disease. However, the exclusion criteria of the present study were more comprehensive than the PNS criteria,^10^ as detailed in the Supplementary Material, resulting in the selection of a healthier population. Additionally, for the calculation of RBC parameters, individuals with hemoglobinopathy were excluded.^10^

 Another difference in relation to the methodology adopted by Rosenfeld et al.^10^ is that our study stratified the RI values only when the parameter presented any of the three conditions recommended by the Harris–Boyd statistic. In contrast, the PNS stratified its entire sample according to sex (male and female), age group (18 to 59 years and 60 years and over), and race/skin color (black, brown, and white). No specific RI by age group was established in our study, as we found no statistical differences with regard to age, possibly because our study included participants aged 35 years or older, whereas the PNS included individuals aged 18 or older.^10^

 The establishment of RIs for laboratory tests is a rigorous and complex process that requires appropriate methodology, adequate sample sizes, and strict standardization and control throughout collection, processing, transport, and analysis. Owing to these challenges, many developing countries adopt parameters from studies conducted in developed nations, which may not accurately represent their populations.^10^ In Brazil, defining population-specific RIs is crucial to improve the clinical interpretation of laboratory results, as these parameters reflect the country’s genetic, environmental, and sociodemographic characteristics. Brazil has a high degree of miscegenation between European, African, and Indigenous ancestry, which influences hematological parameters such as hemoglobin levels, MCV, and leukocyte count. Furthermore, environmental differences (greater exposure to parasites, varying levels of pollution, and dietary habits) can modulate hematopoiesis and immune responses. Therefore, these results reflect the biological characteristics of the Brazilian adult population and reinforce the need to avoid the exclusive use of international reference values.^17-21^

 The adoption of the RIs proposed in this study does not imply immediate changes in clinical practice because therapeutic decision points for conditions such as anemia, leukopenia, or thrombocytopenia are based on consolidated clinical guidelines. However, establishing specific RIs for the Brazilian population is fundamental for improving analytical quality and laboratory standardization and reducing inconsistencies between laboratories that currently use values from foreign situations or outdated methodologies. Although it does not alter the already defined clinical protocols, the use of national RIs improves the accuracy of result interpretation, favoring alignment with the biological characteristics of the Brazilian adult population and strengthening quality control and laboratory accreditation processes. Thus, the main practical implication is the advancement of analytical reliability rather than the immediate redefinition of clinical diagnoses.^21^

 The use of national RIs enhances diagnostic precision, strengthens standardization and quality control across laboratories, reduces interlaboratory variability, and ensures that acceptance limits align with local biological realities. Furthermore, such parameters support the creation of technical standards and public health policies, fostering uniformity in accreditation processes and promoting safer and more effective clinical practices nationwide.^22^

 The limitation of the present study is that it only analyzed data from healthy adults (35–74 years old); no data was collected for adolescents, children, or other special groups such as pregnant women. Some of the exclusion criteria were self-reported, so that participants with diseases unknown to them could have been included in the study. Outliers were excluded to minimize this problem. The strengths of this study include the fact that the sample comprised healthy individuals, enabling a more appropriate analysis to establish RIs. Furthermore, although ELSA-Brasil is not representative of Brazilian adults, it consists of adults from three regions of Brazil, which adds diversity to the sample. 

## CONCLUSION

 Our results are similar to those of previous studies. We believe that by defining specific RIs for our study population, we can obtain more reliable information on the real-world health status of the Brazilian population and contribute to better clinical care and disease control. 

## Data Availability

Data supporting the findings of this study are available from the corresponding author, Danyelle Romana Alves Rios, upon request.
