# Supplementary material for: Reference intervals for complete blood count parameters in the Longitudinal Study of Adult Health (ELSA-Brasil): a cross-sectional analysis
Source: Sao Paulo Med J. 2026 May 22;144(2):e20253017. doi: 10.1590/1516-3180.2025.3017.09122025 (PMC13200561; doi:10.1590/1516-3180.2025.3017.09122025)
Supplement: supplementary material [file 1806-9460-spmj-144-02-e20253017-suppl1.docx]

| **Table S1: Exclusion criteria for participants, N (15,105).** | | |
| --- | --- | --- |
| **Exclusion criteria (n = 15,105)** | **Excluded** | **Included** |
| Missing blood count data | 95 | 15,010 |
| Health problems in the last 12 hours | 1,104 | 13,906 |
| C-reactive protein > 10 | 563 | 13,343 |
| Taking any regular medication | 7,733 | 5,610 |
| State of health (fair, poor, very poor) | 690 | 4,920 |
| Self-reported medical diagnosis of diabetes mellitus | 54 | 4,866 |
| Self-reported medical diagnosis of hypertension | 401 | 4,465 |
| Self-reported medical diagnosis of cardiovascular disease^a^ | 96 | 4,369 |
| Self-reported medical diagnosis of thrombosis or pulmonary embolism | 36 | 4,333 |
| Self-reported medical diagnosis of liver disease^b^ | 336 | 3,997 |
| Cancer | 76 | 3,921 |
| Current smoke^c^ | 536 | 3,385 |
| Body mass index (BMI) ≥ 30 kg/m^2^ | 484 | 2,901 |
| Glomerular filtration rate (GFR) < 60 mL/min/1.72 m^3^ | 50 | 2,851 |
| Exclusion of extreme hematological parameters (outliers)^d^ | 434 | 2,417 |
| **Total** | **2,417** | |
| ^a^Acute myocardial infarction, angina, congestive heart failure, stroke and myocardial revascularization; ^b^cirrhosis or hepatitis; ^c^participants who declared that they had smoked at least 100 cigarettes over the course of their lives and that they were still smoking; ^d^outliers excluded after evaluation of the 1^st^ and 99^th^ percentiles. | | |

| **Table S2.** Comparison of reference intervals for complete blood count parameters in different studies. | | | | | | | | |
| --- | --- | --- | --- | --- | --- | --- | --- | --- |
| **Parameter** | **Female** | | | **Male** | | | **All** | |
|  | ELSA-Brasil^a^ | PNS^b^ | PNCQ^c^ | ELSA-Brasil^a^ | PNS^b^ | PNCQ^c^ | ELSA-Brasil^a^ | PNCQ^c^ |
| RBC (× 10^6^/mm^3^) | 3.9–5.1 | 3.9–5.1 | 3.8–4.8 | 4.4–5.6 | 4.3–5.8 | 4.5–5.5 | 4.0–5.6 | - |
| Hemoglobin (g/dL) | 11.8–14.9 | 11.5–14.9 | 12.0–15.0 | 13.2–16.7 | 13.0–16.9 | 13.0–17.0 | 12.1–16.5 | - |
| Hematocrit (%) | 35.3–44.2 | 35.3–46.1 | 36.0–46.0 | 39.0–49.0 | 39.7–52.0 | 40.0–50.0 | 36.0–48.5 | - |
| MCV (fL) | - | 81.0–100.2 | - | - | 81.8–100.6 | - | 79.9–96.0 | 83.0–101.0 |
| MCH (pg) | - | 26.3–32.4 | - | - | 26.9–32.6 | - | 26.4–32.5 | 27.0–32.0 |
| MCHC (g/dL) | 32.0–35.1 | 30.5–34.3 | - | 32.3–35.7 | 30.6–34.6 | - | 32.0–35.5 | 31.5–34.5 |
| WBC (/mm^3^) | - | 2,883.0–9,969.0 | - | - | 2,843.0–9,440.0 | - | 3,700.0–8,610.0 | 4,000.0–10,000.0 |
| Neutrophils (/mm^3^) | - | 612.0–6,474.0 | - | - | 576.0–5,971.0 | - | 1,666.0–5,705.0 | 2,000.0–7,000.0 |
| Eosinophils (/mm^3^) | - | 0.0–550.0 | - | - | 0.0–660.0 | - | 23.8–530.0 | 20.0–500.0 |
| Basophils (/mm^3^) | - | 0.0–72.0 | - | - | 0.0–62.0 | - | 0.0–112.0 | 20.0–100.0 |
| Lymphocytes (/mm^3^) | - | 796.0–3,414.0 | - | - | 720.0–3,370.0 | - | 1,121.0–2,824.0 | 1,000.0–3,000.0 |
| Monocytes (/mm^3^) | - | 22.0–692.0 | - | - | 11.0–812.0 | - | 240.0–751.4 | 200.0–1,000.0 |
| Platelets (× 10^3^/mm^3^) | 170.0–352.0 | 135.6–343.0 | - | 146.0–319.0 | 128.0–299.7 | - | 151.0–339.0 | 150.0–400.0 |
| ^a^ELSA-Brasil: Longitudinal Study of Adult Health - Brazil; ^b^PNS: National Health Survey; PNCQ: National Quality Control Program; ^c^The RI of the complete blood count parameters provided by the National Quality Control Program (PNCQ), which uses as a reference the values included in Bain JB, Bates I and Mike A. Dacie and Lewis - Practical Haematology. 12th Edition, 2017. RBC: red blood cells; MCV: mean corpuscular volume; MCH: Mean Corpuscular Hemoglobin; MCHC: Mean Corpuscular Hemoglobin Concentration; WBC: white blood cells. | | | | | | | | |
